# Mechanism of skeletal muscle atrophy after spinal cord injury: A narrative review

**DOI:** 10.3389/fnut.2023.1099143

**Published:** 2023-03-03

**Authors:** Xin Xu, Zuliyaer Talifu, Chun-Jia Zhang, Feng Gao, Han Ke, Yun-Zhu Pan, Han Gong, Hua-Yong Du, Yan Yu, Ying-Li Jing, Liang-Jie Du, Jian-Jun Li, De-Gang Yang

**Affiliations:** ^1^School of Rehabilitation, Capital Medical University, Beijing, China; ^2^Department of Spinal and Neural Functional Reconstruction, China Rehabilitation Research Center, Beijing, China; ^3^Chinese Institute of Rehabilitation Science, Beijing, China; ^4^Center of Neural Injury and Repair, Beijing Institute for Brain Disorders, Beijing, China; ^5^Beijing Key Laboratory of Neural Injury and Rehabilitation, Beijing, China; ^6^School of Rehabilitation Sciences and Engineering, University of Health and Rehabilitation Sciences, Qingdao, Shandong, China

**Keywords:** spinal cord injury, skeletal muscle atrophy, denervation, immobilization, inflammation, oxidative stress

## Abstract

Spinal cord injury leads to loss of innervation of skeletal muscle, decreased motor function, and significantly reduced load on skeletal muscle, resulting in atrophy. Factors such as braking, hormone level fluctuation, inflammation, and oxidative stress damage accelerate skeletal muscle atrophy. The atrophy process can result in skeletal muscle cell apoptosis, protein degradation, fat deposition, and other pathophysiological changes. Skeletal muscle atrophy not only hinders the recovery of motor function but is also closely related to many systemic dysfunctions, affecting the prognosis of patients with spinal cord injury. Extensive research on the mechanism of skeletal muscle atrophy and intervention at the molecular level has shown that inflammation and oxidative stress injury are the main mechanisms of skeletal muscle atrophy after spinal cord injury and that multiple pathways are involved. These may become targets of future clinical intervention. However, most of the experimental studies are still at the basic research stage and still have some limitations in clinical application, and most of the clinical treatments are focused on rehabilitation training, so how to develop more efficient interventions in clinical treatment still needs to be further explored. Therefore, this review focuses mainly on the mechanisms of skeletal muscle atrophy after spinal cord injury and summarizes the cytokines and signaling pathways associated with skeletal muscle atrophy in recent studies, hoping to provide new therapeutic ideas for future clinical work.

## Introduction

Spinal cord injury (SCI) is a serious and disabling disease. In recent years, the incidence of spinal cord injury caused by traffic accidents, industrial accidents, and sports injuries has been increasing (1–4). SCI leads to the loss of central regulation of peripheral nerves below the injured segment, resulting in sensory, motor, and autonomic dysfunction, muscle paralysis, and reduced muscle load (5, 6).

Injury to the neuromuscular system and reduced integrity of the musculoskeletal system are important features of SCI (7), and are also significant obstacles to the recovery of motor function. As the main component of human tissue structure, skeletal muscle accounts for about 40% of body weight (8). In addition to maintaining the homeostasis of exercise, skeletal muscle also plays a number of physiological functions such as support, protection, and respiration (9, 10). The muscle type most frequently atrophied after SCI is skeletal muscle, manifesting as loss of mass and strength (8), with 18–46% decreases in cross-sectional area (CSA) of skeletal muscle 6 weeks after injury (7, 11, 12).

After SCI, somatic and visceral nerve functions are affected (4), and these strongly correlate with skeletal muscle atrophy. After somatic nerve function damage, the corresponding skeletal muscle is denervated, and motor dysfunction is severe in patients with higher injury segments. The limbs of these patients maintain long-term braking and lose the nutritional effect of the nerve, causing skeletal muscle physiological, biochemical and biomechanical changes, and many functional reductions (13–15). Visceral nerve function damage leads to disruption of some hormones secretion, many of which are closely related to the maintenance of skeletal muscle mass, such as testosterone, insulin, growth hormone, and others (16–18), with multiple factors coinciding to aggravate skeletal muscle atrophy after SCI (19). Skeletal muscle atrophy can cause systemic secondary metabolic dysfunction, such as glucose intolerance, type 2 diabetes and insulin resistance (6, 20). Although the quality and function of skeletal muscles can be recovered to some extent through voluntary movement, the effect is often very limited (21) and is easily affected by the patient's psychological focus and other factors, and the optimal recovery period may be missed. Muscle atrophy is irreversible even if the axons, endplates, and skeletal muscle can be reconnected later (22). Therefore, maintaining skeletal muscle integrity is crucial for maintaining cell homeostasis and systemic metabolism, and research on the prevention and treatment of skeletal muscle atrophy after SCI is highly significant for optimal patient benefit (23, 24).

In recent years, there have been many studies on skeletal muscle atrophy after spinal cord injury, but most of them are experimental studies aimed at prevention and treatment strategies, but much work is still needed before practical clinical application. There is also a lack of review of the related mechanisms of skeletal muscle atrophy. However, it is important to clarify and organize the factors, pathological changes and related mechanisms of skeletal muscle atrophy after spinal cord injury for subsequent research and treatment.

## Factors in skeletal muscle atrophy

**Figure 1:**
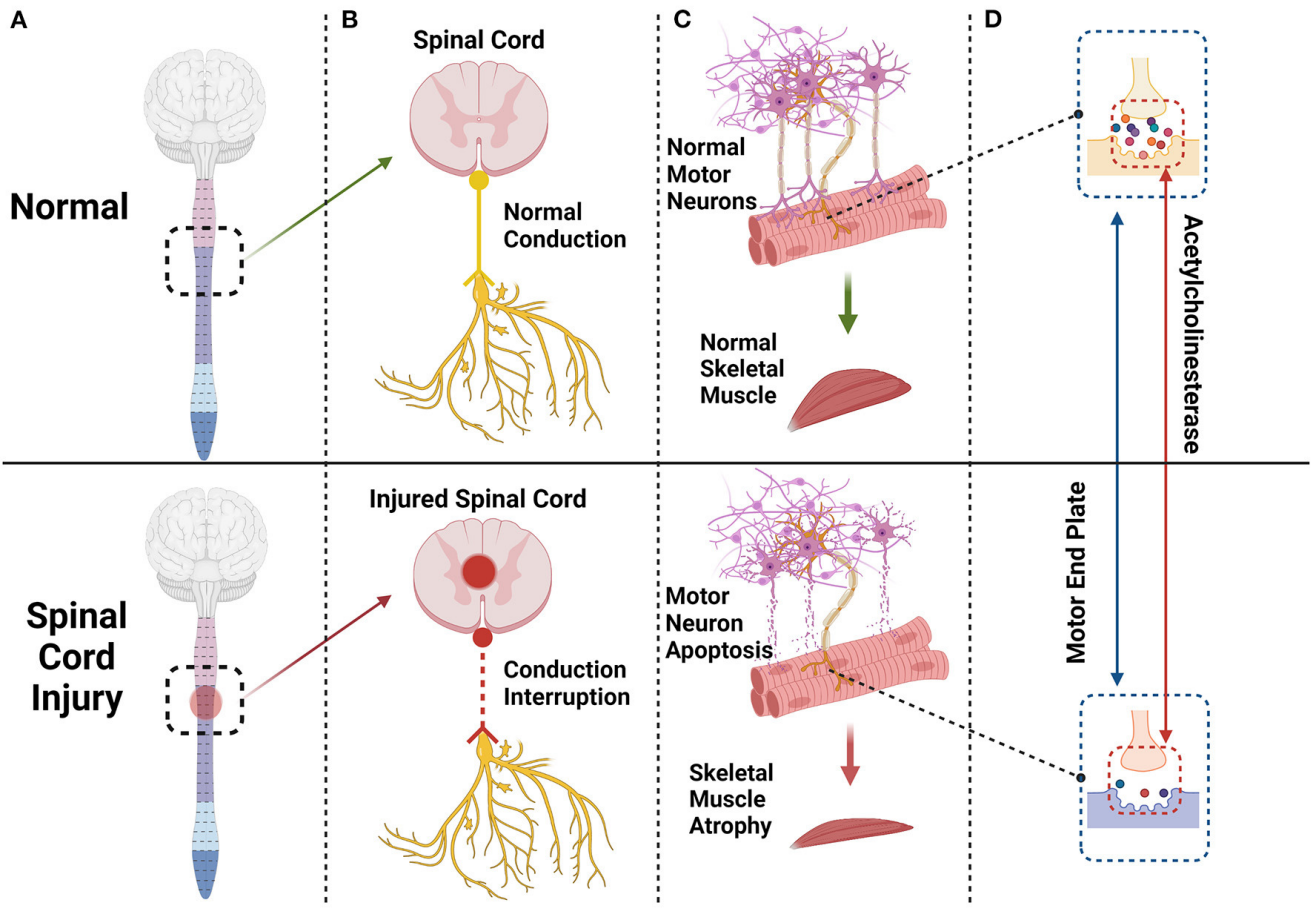
The spinal cord and brain constitute the central nervous system. After a spinal cord injury **(A)** the conduction of peripheral nerves innervating skeletal muscle will be interrupted or blocked **(B)** and some motor neurons innervating skeletal muscle will undergo apoptosis **(C)** Endplates degenerate, and acetylcholinesterase content of synapses will decrease significantly **(D)** eventually leading to skeletal muscle atrophy **(C)**.

SCI often results in upper or lower motor neuron damage or both (7), upper being relatively common, and the muscle atrophy differs between the two (25). When upper motor neurons are injured, the lower motor neurons are often intact. In SCI the conduction process is blocked (26) (Figure 1). Spinal motor neurons become highly excited (27), thereby pathologically activating the antigravity muscles of the lower limbs, such as the quadriceps femoris, gastrocnemius, and others, leading to spasticity. Deep hyperreflexia occurs (28), but moderate spasticity also reduces muscle atrophy to some extent (29, 30).

Although the signs of muscle atrophy after SCI are standard in most forms of disuse muscle atrophy, skeletal muscle atrophy is often faster after SCI due to denervation, immobilization, and other factors (23). This is because the γ-loop is damaged after spinal cord injury (31), leading to inhibition of α motor neurons that excite muscles, accompanied by different levels and patterns of muscle atrophy signals and anabolic signals. A combination of factors caused rapid skeletal muscle atrophy after SCI (20). Severe SCI results in impaired neural drive function, accompanied by changes in neuromuscular junctions (8, 20), and leads to impaired skeletal muscle function around and below the injury site. Etzel et al. (32) found in rats that compared with simple immobilization, SCI resulted in a more significant decrease in muscle cross-sectional area, wet muscle weight, and muscle strength within 7–21 days after immobilization. This indicates that neural input and mechanical load have a combined effect on the mass and strength of skeletal muscle after SCI.

## Pathophysiological process

**Figure 2:**
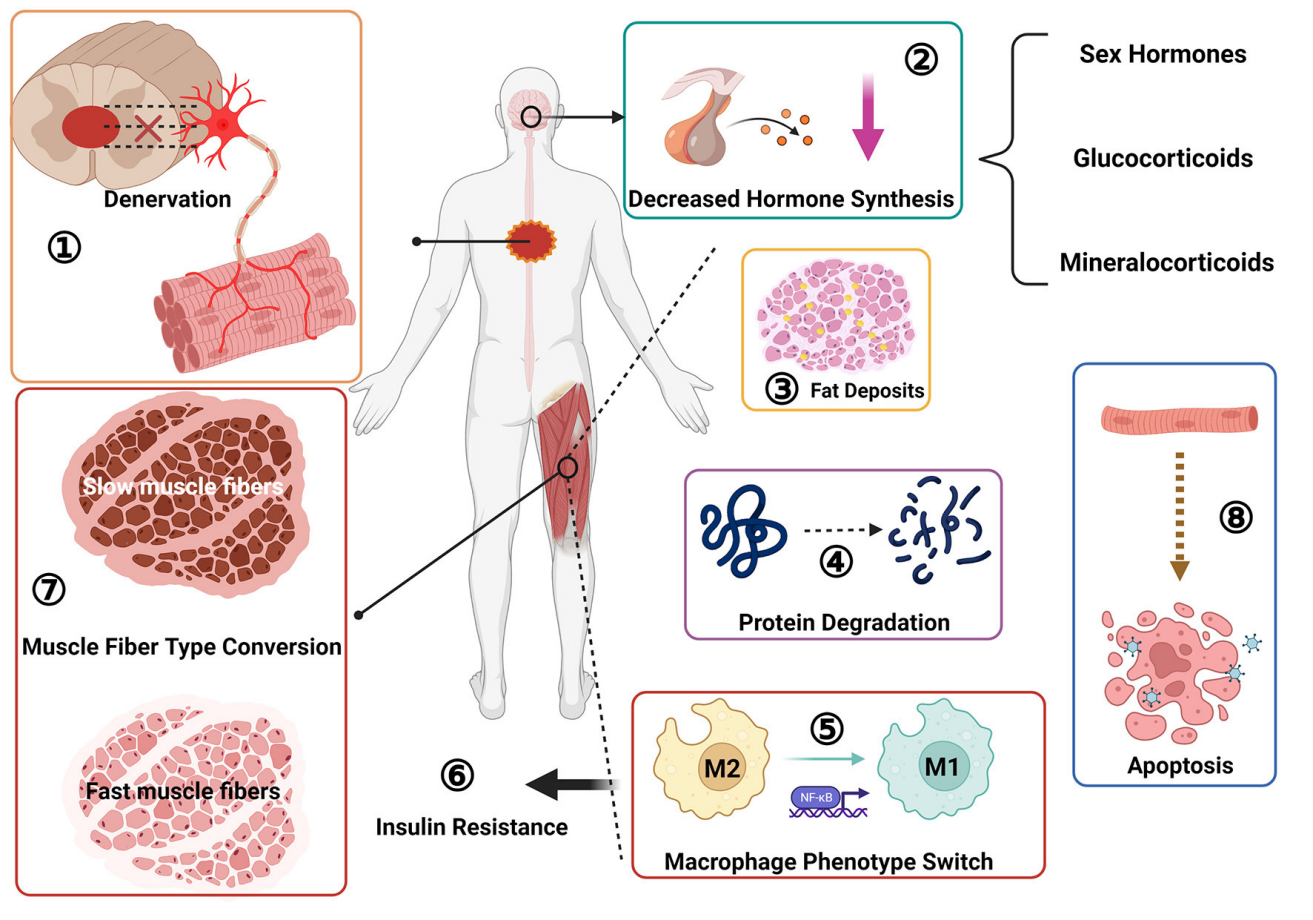
Pathological changes of skeletal muscle after spinal cord injury: ① muscle denervation, neuromuscular junction degeneration; ② decreased sex hormone secretion; ③ fat deposition; ④ protein degradation; ⑤ macrophage phenotype switch, from M2 to M1 type transition; ⑥ insulin resistance; ⑦ muscle fiber type transition, from slow oxidation type to fast glycolysis type; ⑧ muscle cell apoptosis.

The spinal cord establishes a nutritional connection with skeletal muscle through peripheral nerves, and spinal motor neurons also trigger the contraction of skeletal muscle by transmitting action potentials to the motor endplate (33). When the spinal cord is injured, some motor neurons undergo apoptosis. The morphology and function of the remaining motor neurons also change; synapses are shortened (34), and the skeletal muscles innervated by these motor neurons and synapses undergo some level of atrophy and fibrosis (35–37). The motor endplate, also known as the neuromuscular junction, is the chemical synapse between motor neurons and skeletal muscle, consisting of motor nerve endings, the synaptic cleft, acetylcholine-containing synaptic vesicles, and a postsynaptic membrane (38). The motor endplate degenerates after injury, and its acetylcholinesterase (AchE) content decreases significantly (Figure 1). Acetylcholine cannot be removed in time, and excess calcium flows into the postsynaptic membrane through acetylcholine receptor channels. Intracellular proteases are activated in skeletal muscle cells, triggering protein degradation and apoptosis (33, 39, 40) (Figure 2).

The protein content of skeletal muscle accounts for about 60% of total body protein (41). Skeletal muscle atrophy after SCI is mainly a process of protein production and degradation imbalance (42). After an injury, protein hydrolysis is activated, the rate of protein degradation is greater than the rate of synthesis (8) and many organelles and muscle contractile proteins are degraded into free amino acids (43–45). Studies have shown that long-term disuse-related muscle atrophy is mainly due to a reduced rate of protein synthesis. In contrast, short-term disuse-related muscle atrophy is accompanied by a reduction in protein synthesis and an increase in decomposition, so the early stage of muscle disuse is manifested as clearly apparent muscle atrophy (46), indicating that short-term disuse-related muscle atrophy is more closely related to muscle mass and volume reduction. A study by Moore et al. (11) showed that muscle CSA in an SCI group decreased compared with that of a control group (8, 11), and the degree of muscle atrophy was closely related to the degree of injury. Muscle atrophy is more pronounced in complete than incomplete SCI (47). Metabolic protein changes have consequences throughout the lifespan, so age can also exacerbate the process of muscle wasting in patients with SCI (46) (Figure 2).

A persistent secondary injury cascade follows SCI (28), including many systemic effects closely related to skeletal muscle atrophy. For example, levels of hormones such as testosterone (a sex hormone), glucocorticoids, mineralocorticoids and others may fluctuate. Studies have found that dihydrotestosterone, the active metabolite of testosterone, may reduce synaptic dissection after nerve injury (35), strengthen afferent central nervous system signals, and promote the recovery of motor function (48). Men are prone to hypotestosteronemia after SCI, and exogenous testosterone treatment can prevent oxidative neural stress damage, effectively preventing SCI skeletal muscle atrophy and maintaining skeletal muscle mass [16]. Studies have found that another sex hormone, estradiol, can improve motor function and has anti-inflammatory effects, effectively reducing apoptosis (35, 49) and preventing further damage to spinal cord tissue. Its main target is the nervous system, but whether estradiol has a direct therapeutic effect on skeletal muscle atrophy after SCI remains unclear. Hormone therapy has shown strong potential in improving motor function and reducing skeletal muscle atrophy. Synthetic steroid hormones are used clinically in treating skeletal muscle atrophy, such as testosterone, insulin-like growth factor, and others. Combating skeletal muscle atrophy by increasing the transcriptional level of myogenic fibronectin DNA and activating the proliferation and differentiation of muscle satellite cells (50) (Figure 2). However, hormone therapy, while having a better therapeutic effect, is also associated with a certain risk of side effects, so it has to be applied appropriately and may not be suitable for all patients, and therefore researchers are currently exploring alternative treatments.

The spinal cord mainly relies on α motor neurons to regulate skeletal muscle, at the same time according to the regulation of different types of muscle fibers, α motor neurons can be divided into S motor neurons (slow contraction, anti-fatigue), FR motor neurons (fast contraction, fatigue-resistant), FF motor neurons (fast contraction, easy fatigue) three subtypes, so as to coordinate the various functions and effects of skeletal muscle, control type I muscle fibers, IIb muscle fibers and IIx muscle fibers respectively (51). Spinal cord injury can cause slow motor neuron axon conduction speed to slow down (52, 53). Skeletal muscle fiber undergoes a transition after SCI from a slow-oxidative to a fast fatigue and fast glycolysis variety, and at the same time develops toward the direction of muscle fibrosis, that is, the transformation of type II fiber to type I fiber (7, 54–57). which often precedes the deposition of adipose tissue (7), this may attribute to changes in the expression of genes that control myosin subunits, resulting in a significant decrease in the proportion of the slow myosin heavy chain (MHC) isoform and a corresponding increase in the fast MHC isoform (58, 59). However, if spasticity occurs after spinal cord injury, the change in muscle fiber type may not be obvious (56) (Figure 2).

In addition to skeletal muscle fiber changes, specific abnormalities occur in the glucose and lipid metabolism of the skeletal muscle. Intramuscular fat (IMF) comprises fat infiltrated within a single muscle group (intramuscular and extra muscular fat compartments) and intermuscular adipose tissue between different muscle groups. An essential pathological change after skeletal muscle atrophy is the deposition and infiltration of numerous IMFs (11) more extensively in patients with SCI than in healthy individuals. Elder found that the IMF content in the muscles of SCI subjects was more than three times that of control subjects, and the subfascial fat content was about four times that of controls, and this is a cause of decreased strength in SCI patients (60) (Figure 2).

The glucose metabolism of skeletal muscle becomes disordered after SCI. Skeletal muscle, as an important consumer of glucose (61), develops insulin resistance (60) and initiates an inflammatory response in skeletal muscle after SCI, with an increase in macrophages within the muscle. The phenotype of muscle macrophages can affect the insulin sensitivity, and macrophages will polarize from the M2 phenotype to the M1 phenotype (62, 63), inducing insulin resistance. Macrophages may also affect the uptake and metabolism of glucose in skeletal muscle by affecting the secretion of factors related to glucose homeostasis (64), also resulting in insulin resistance in the muscle. These changes account for the occurrence of type 2 diabetes mellitus after SCI (65, 66) (Figure 2).

## Cytokines associated with skeletal muscle atrophy after SCI

Skeletal muscle atrophy is often closely related to the severity of spinal cord injury. Incomplete spinal cord injury tends to cause skeletal muscle atrophy within the first 6 weeks of injury (6), while complete spinal cord injury continues to cause skeletal muscle atrophy within 24 weeks after injury (7). Chronic inflammatory response and oxidative stress in skeletal muscle after SCI may be the mechanisms leading to atrophy (9, 67–69). At present, the known factors and related proteins involved in skeletal muscle atrophy include: tumor necrosis factor-alpha (TNF-α) and its receptor (70–72), human tumor necrosis factor-related weak apoptosis-inducing factor (TWEAK) and its receptor (56, 67, 73), interleukin-1β (IL-1β) (72, 74), interleukin-6 (IL-6) and its receptor (75, 76), growth factor (IGF-1) (77, 78), human dystrophin (Fbox-1, also known as Atrogin-1) (79, 80), muscle-specific RING finger protein 1 (MuRF1) (79, 80), peroxisome proliferator-activated receptor-γ coactivator-1α (PGC-1α) (20, 58, 81), fibroblast growth factor-inducible receptor 14 (Fn14) (73), reactive oxygen species (ROS) (20, 68, 82) and others. The discovery of these skeletal dystrophins has provided a deeper understanding of skeletal muscle atrophy at the molecular level and suggest the possibility of intervening *via* corresponding signaling pathways and factors to delay the atrophy process or promote skeletal muscle regeneration.

## Inflammation-mediated skeletal muscle atrophy after spinal cord injury

### Tumor necrosis factor-alpha

**Figure 3:**
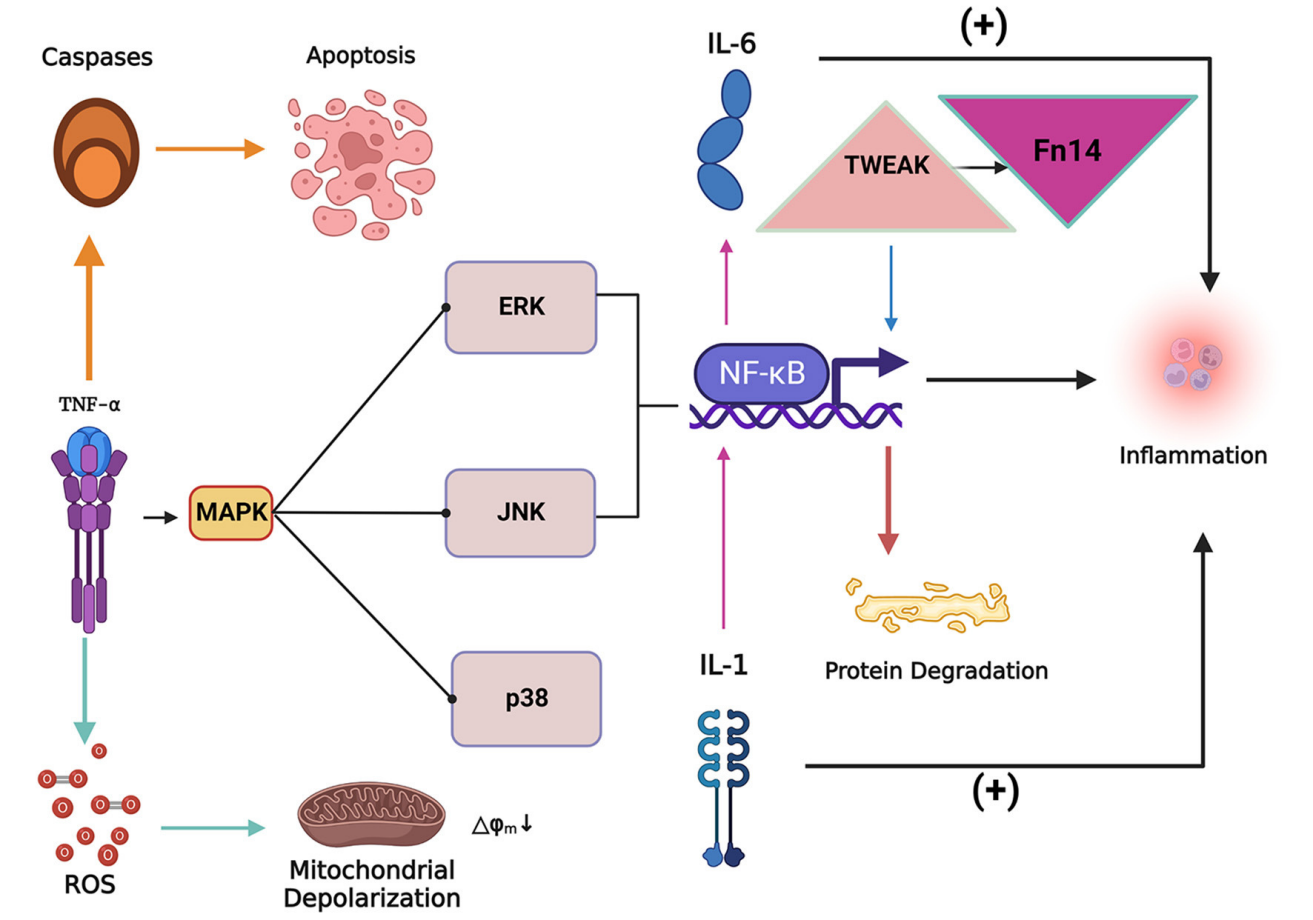
The inflammatory factor tumor necrosis factor-alpha (TNF-α) can induce the apoptosis of skeletal muscle cells and lead to the accumulation of reactive oxygen species (ROS). The collection of ROS can lead to mitochondrial dysfunction and depolarization, while TNF-α can activate the MAPK signaling pathway and cause nuclear factor kappa-light-chain-enhancer of activated B cells (NF-κB). NF-κB expression levels to increase, inducing inflammation and protein degradation. Tumor necrosis factor-related weak apoptosis-inducing factor (TWEAK) can activate NF-κB signaling to promote proteolysis and simultaneously increase the expression level of fibroblast growth factor-inducing factor 14 (Fn14), both of which act synergistically in the process of skeletal muscle atrophy. IL-1 (Interleukin 1) and IL-6 (Interleukin 6) are critical inflammatory factors that induce skeletal muscle atrophy. IL-1 stimulates the expression of IL-6 by stimulating the expression of NF-κB. Overexpression of IL-1 and IL-6 induces an inflammatory response in the body and thus induces skeletal muscle atrophy.

As a pro-inflammatory cytokine, TNF-α is a potent inducer of skeletal muscle atrophy after SCI. It was found that the expression levels of TNF-α and TNF-α receptors were elevated in the atrophied skeletal muscle during the chronic phase of spinal cord injury patients, which played a very important role in mediating skeletal muscle atrophy during the chronic phase of SCI (5, 67, 72). TNF-α binding to tumor necrosis factor receptor 1 (TNFR1) induces atrophy and autophagy of C2C12 myotubes in skeletal muscle (9, 42), while leading to ROS accumulation and activating the inflammatory response pathway. The expression level of nuclear factor kappa-light-chain-enhancer of activated B cells (NF-κB) is also increased, and the degradation rate of skeletal muscle protein is accelerated (83, 84). TNF-α is closely related to the occurrence of energy disorder and stress as well as abnormal glucose and lipid metabolism in the process of skeletal muscle atrophy (42). Consistent with this, its levels are significantly positively correlated with muscle strength (85). The MAPK signaling pathway is activated to varying degrees after SCI due to various factors such as oxidative stress and inflammation (86, 87). TNF-α as an inflammatory factor activates the MAPK signaling pathway mediated by extracellular regulated protein kinas (ERK), p38 MAPK, c-Jun aminoterminal kinase (JNK) mediated MAPK signaling pathway (83, 88) (Figure 3) and enhances the expression of Atrogin-1 and MuRF1.

Previous studies have found that the differentiation of C2C12 myoblasts in the early stage of skeletal muscle injury is strongly correlated with the concentration of TNF-α (89), which is closely related to the tissue repair effect of TNF-α in the acute phase, while in the chronic phase TNF-α is related to tissue damage (90). Low doses of TNF-α can induce the proliferation of C2C12 myoblasts, promote muscle production in skeletal muscle cells, while high doses cause skeletal muscle atrophy by interfering with the ability of muscle cells to differentiate into muscle fibers. Therefore, TNF-α exhibits a time- and dose-dependent induction of skeletal muscle atrophy (42). It was found that functional electrical stimulation therapy and endurance exercise training can effectively reduce the level of TNF-α in SCI patients and animals, thus acting as a treatment against skeletal muscle atrophy (91, 92). Therefore, suppressing elevated levels of inflammatory factors in the acute phase of SCI or fighting chronic inflammation in the chronic phase are also effective strategies to treat skeletal muscle atrophy.

### Tumor necrosis factor-like weak inducer of apoptosis /fibroblast growth factor-inducing factor 14

TWEAK may be an essential mediator of chronic inflammation and fibrotic changes in skeletal muscle after SCI, and higher levels of TWEAK and TWEAK R in SCI may affect the oxidative metabolism of the body. On the one hand, TWEAK inhibits the normal oxidative metabolic process in skeletal muscle by activating NF-κb, thus causing oxidative stress injury (67, 93). On the other hand, TWEAK can activate NF-κB signaling and other proteolytic pathways (94) (Figure 3), activate the autophagy pathway, induce muscle proteolysis, and inhibit the proliferation of myoblasts, thereby inhibiting the regeneration of skeletal muscle fibers. In addition, TWEAK reduces the number of mitochondria in skeletal muscle cells, weakens the ability of skeletal muscle cells to resist oxidative stress (93), and causes metabolic dysfunction in skeletal muscle cells (93, 95).

Fn14 is stimulated and up-regulated after SCI, and can combine with TWEAK to synergistically affect the atrophy of skeletal muscle (93) (Figure 3). It may also be involved in the transition process of skeletal muscle fiber types (93), but at higher levels Fn14 has a TWEAK-independent effect, promoting the expression of critical factors in skeletal muscle regeneration, thereby promoting skeletal muscle regeneration (94). Activation of TWEAK/Fn14 is also coupled to TNF-α-TNFR1 signaling and can sensitize skeletal muscle to TNF-α signaling (96).

In the persistent state of injury after SCI, TWEAK-TWEAK R activation may lead to pathological remodeling of muscle, activating proliferation and activation of fibroblasts and causing muscle fibrosis. Yarar-Fisher et al. (67) found that expression levels of TWEAK R, Fn14, and NK-κB in the skeletal muscle of patients with SCI were significantly increased, and the degree of muscle fibrosis was also significantly increased, suggesting a close association. In addition, TWEAK is also an important regulator of the skeletal muscle fiber type transition process after SCI (67). Mittal (55) and others compared mice with overexpression or knockout of the TWEAK gene and found that skeletal muscle atrophy and fast muscle fibers increased significantly after 4 to 5 months of overexpression, with increased Fn14 expression level and the opposite finding in the knockout group. These studies have confirmed that the TWEAK/TWEAK R/NF-κB signaling pathway and Fn14 play essential roles in skeletal muscle atrophy after SCI.

### Interleukin 1/6

SCI activates the body's immune and inflammatory responses, causes an increase in interleukin levels, such as IL-1β, IL-6, and IL-1β causes secondary spinal cord injury, which further leads to loss of central regulation of skeletal muscle, also IL-1β has a role in promoting neurogenic heterotopic ossification (NHO) in skeletal muscle after SCI, which limits joint movement and affects the normal life of patients (97–100). IL-6 is mainly produced during skeletal muscle contraction (101). High levels of IL-6 are also an important influence on skeletal muscle atrophy after SCI (102).

Satellite cells are the power source for skeletal muscle cell proliferation and regeneration, maintain skeletal muscle quality, and endow skeletal muscle with a degree of plasticity (103). IL-1 and IL-6 have pro-inflammatory effects, activating satellite cells in skeletal muscle, promoting muscle cell proliferation to some extent, and have a particularly positive impact on promoting muscle regeneration in the early stage of injury (99, 104). However, high levels of IL-1 and IL-6 combined with TNF-α can inhibit the synthesis and metabolism of IGF-1 (69). At an appropriate level, IL-1β can promote the expression of cyclooxygenase 2 (COX-2) in muscle and reduce myostatin levels (103). A study (105) showed that the proliferation and differentiation of myoblasts was reduced in IL-1 knockout mice. With low concentrations of exogenous IL-1 introduced to the body, the MAPK signaling pathway was activated, and NF-κB stimulated the secretion of chemokines and IL-6 (Figure 3). Low concentrations of IL-6 have a positive effect on the promotion of muscle cell proliferation by IL-1β. However, when spinal cord injury enters the chronic inflammatory phase, it has the opposite effect, mainly by activating the ubiquitin-proteasome system, inducing skeletal muscle atrophy (9, 106), and simultaneously generating the production of myostatin and causing cell damage. The accumulation of mitochondrial ROS (84, 107) and other factors induce skeletal muscle atrophy.

Skeletal muscle is an integral producer of IL-1 and IL-6 (97), healthy individuals can promote IL-6 levels through exercise, but under short-term exercise SCI patients lack such a regulatory mechanism due to skeletal muscle atrophy (101), and studies have found that endurance exercise can regulate the expression of IL-1 and IL-6 in skeletal muscle (103, 108), which may explain the positive effect of endurance training on skeletal muscle function and muscle strength after SCI (75, 109).

## Oxidative stress-mediated skeletal muscle atrophy after spinal cord injury

### Peroxisome proliferator-activated receptor-γ coactivator (PGC)-1α

PGC-1α is a regulator of mitochondrial bioenergetics and abundantly expressed in skeletal muscle, and plays an essential role in skeletal muscle repair after SCI *via* transformation of fast-twitch to slow-twitch muscle fibers (77, 81, 110, 111). It can induce mitochondrial biogenesis, and by up-regulating nuclear respiratory factors 1,2 (Nrf1, 2) and mitochondrial transcription factors expressed against oxidative stress in skeletal muscle and spinal cord (81, 112) it can increase myoglobin activity and promote energy metabolism in skeletal muscle. After SCI, PGC-1α expression decreases (110), and expression of myosin heavy chain protein also decreases accordingly (31). PGC-1α has an inhibitory effect on FoxO_3_ associated with muscle atrophy and mass loss (110)whose expression is increased after SCI, indirectly causing the expression of Atrogin-1 and MuRF1, resulting in muscle atrophy (113). It was found that exercise training promotes the expression of PGC-1α and the transformation of spinal cord injury patients' muscle fibers from fatigue-prone fast muscle fibers to more endurance-prone slow muscle fibers, while improving muscle endurance (111).Therefore, PGC-1α plays a vital role in the repair of nerve and skeletal muscle structure and function after SCI.

### Reactive oxygen species

**Figure 4:**
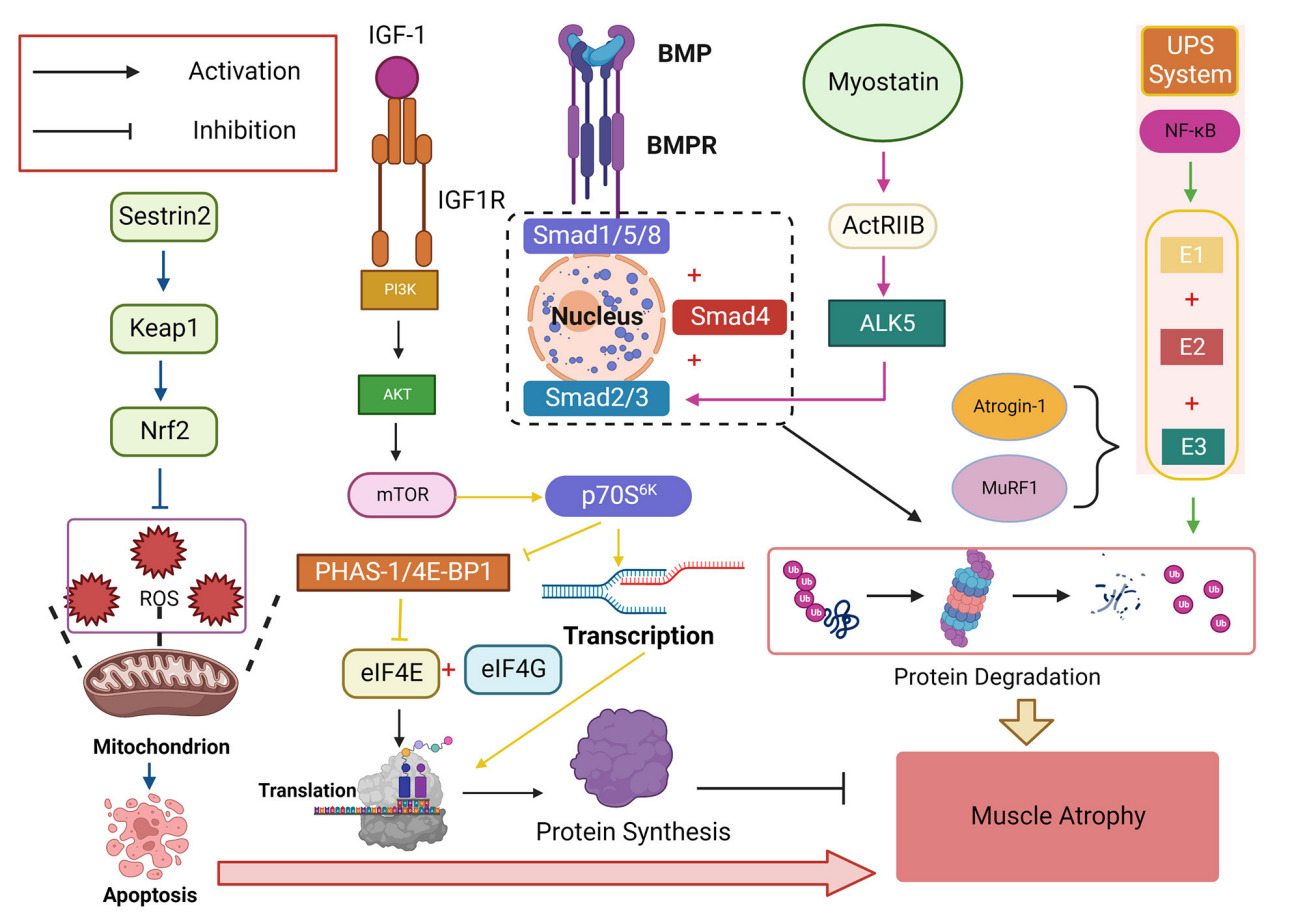
The insulin-like growth factor-1 (IGF-1)/phosphatidylinositol trikinase (PI3K)/threonine kinase (Akt)/mammalian target of rapamycin (mTOR) signaling pathway is a critical signaling pathway that promotes protein synthesis and plays a vital role in regulating skeletal muscle mass. Its downstream target p70S6K can promote protein transcription while inhibiting eIF4E, a negative regulator of PHAS-1/4E-BP1, which binds eIF4E to eIF4G and initiates translation. NF-κB activates ubiquitin-proteasome System (UPS) and degrades proteins by binding E1, E2, and E3, with Atrogin-1 and muscle-specific RING finger protein 1 (MuRF1) being the two most common E3s Ligase, bone morphogenetic protein (BMP) affects the signaling pathway of Smad1, Smad5, and Smad8 (Smad1/5/8) protein phosphorylation and finally converges to Smad4. Myostatin-ActRIIB-ALK5-Smad2/3 is another signaling pathway that finally combines to Smad4 to cause protein degradation in skeletal muscle. Sestrin2 can activate the Sestrin2-Keap1-Nrf2 pathway, increase the expression level of Nrf2, resist ROS-induced apoptosis, and play an antioxidant role.

Changes in mitochondrial structure and function in skeletal muscle cells play a vital role in regulating overall skeletal muscle mass and function (93). Denervation of skeletal muscle after SCI can lead to mitochondrial toxicity (68, 114), this leads to oxidative stress damage, mainly in the form of an imbalance between ROS production and detoxification (68). After the mitochondrial function is damaged, intracellular ROS cannot be effectively and promptly removed. The antioxidant capacity of skeletal muscle cells decreases, resulting in oxidative stress and ROS accumulation, causing oxidative stress damage, apoptosis and mitochondrial depolarization of myoblasts (Figures 3, 4) (115, 116), so scavenging ROS and alleviating oxidative stress damage is an integral part of the treatment of skeletal muscle atrophy after SCI. Under normal circumstances, ROS scavenging mainly depends on antioxidant-related enzymes and factors *in vivo*, such as catalase, glutathione, superoxide dismutase, and others (41, 117, 118). The antioxidant capacity of the spinal cord and skeletal muscle is impaired after SCI, so exogenous interventions are often required to reduce or reverse oxidative stress injury (119). Studies have found that many vitamins, proteins, and other nutrients have antioxidant capacity. For example, vitamin D, especially its 1,25-(OH)2D form, is generally considered to be an antioxidant and its supplementation can reduce skeletal muscle, illustrating the potential for ROS generation to combat oxidative stress (120).

### Sestrins (Sesns)

Sestrins is a highly conserved stress-inducible protein family, which has attracted the attention of researchers in recent years due to its anti-aging and muscle atrophy effects. It can activate AKT and then autophagy, restoring neuronal autophagy flux. It can also activate the MAPK signaling pathway to combat apoptosis and oxidative stress, can affect the homeostasis of stem cells in skeletal muscle (121–125), combat pathological changes such as insulin resistance and fat accumulation (126), and coordinate skeletal muscle synthesis and catabolism to delay the disuse of muscle atrophy (127).

Sestrin1 has the highest expression level in skeletal muscle, and decreases rapidly after SCI, while overexpression of the Sestrin1 gene may reduce atrophy of skeletal muscle (127, 128). SCI can also activate the Sestrin2-Keap1-Nrf2 pathway, increase the expression level of Nrf2, enhancing its antioxidant effect (126) (Figure 4). Elevated sestrin2 levels were found to improve functional recovery and neuronal survival after spinal cord injury through activation of autophagy (122). Therefore, the Sestrins protein family significantly improves SCI skeletal muscle function and promotes structural recovery, and should be further explored as a potential therapeutic target.

## Other related factors regulating skeletal muscle atrophy after spinal cord injury

### Insulin-like growth factor-1

IGF-1 is expressed in skeletal muscle where it plays a vital role in muscle and nerve metabolism. IGF-1 can regulate the proliferation and differentiation of muscle satellite cells by activating PI3K/Akt (77, 129, 130) and promote collagen formation, in turn promoting skeletal muscle hypertrophy (131). It also has a specific nutritional effect on the nervous system, enabling the protein synthesis of neurons and glial cells, inhibiting apoptosis, promoting nerve regeneration and myelination (132), and strengthening the nervous system and skeletal muscle connection. However, IGF-1 is highly sensitive to the inflammatory response. Cheng et al. (133) found that the inflammatory response after SCI resulted in decreased IGF-1 levels in skeletal muscle, which caused diminished anabolism of muscle tissue and atrophy of skeletal muscle (23, 134).

#### Hypothalamus-growth hormone (GH)-IGF-1 axis

GH plays a role in denervated muscle regeneration after SCI by inducing the production of IGF-1 in the liver (132). In chronic SCI patients, the (GH)-IGF-1 axis is inhibited (77, 135). The main reason may be that chronic inflammation caused by SCI leads to increased expression of pro-inflammatory cytokines, stimulates the hypothalamus to secrete growth Inhibitors, inhibits the hypothalamus-GH-IGF-1 axis, and increases GH resistance. Some studies have proposed that inflammatory factors work by inhibiting GH signal transduction (132, 134). For example, TNF-α and IL-1β can inhibit the abundance of growth hormone receptors, while IL-6 plays a role in promoting the expression of suppressor of cytokine signaling 3 (SOCS3), which has an enhancing effect on GH resistance (134).

#### IGF-1/PI3K/Akt/mTOR signaling pathway

IGF-1/phosphatidylinositol trikinase (PI3K)/threonine kinase (Akt)/mammalian target of rapamycin (mTOR) is a signaling pathway that promotes protein synthesis and is involved in the regulation of bone. It plays a vital role in muscle mass (77, 131, 136) (Figure 4). This signaling pathway is inhibited after SCI, and reactivation can resist the resulting skeletal muscle atrophy (137–139) and inflammatory response (133). The PI3K/Akt/mTORC1 pathway plays an essential role in the recovery of denervated skeletal muscle (107).

PI3K is located in the hypothalamus and is anti-flammatory after SCI, and activation of this kinase can be used primarily to counteract the inflammatory response, which has a protective effect in preventing the development of insulin resistance after SCI (133). Akt/mTOR regulates skeletal muscle cell proliferation and growth, normal protein metabolism, and prevents skeletal muscle atrophy. This signaling pathway is down-regulated in skeletal muscle atrophy (41, 137). After SCI, the PI3K/Akt/mTOR pathway is inhibited, and the skeletal muscle protein degradation program is initiated (50). IGF-1 creates this signaling pathway through PI3K and Akt kinases *in vivo*, activating mTOR and phosphorylating it. mTOR is sensitive to changes in amino acids and is a critical protein turnover regulator that integrates nutrition signals, growth factors, energy status, and stress (19, 140). Amino acid-sensitive signals converge on GTPases, which are immobilized on the surface of lysosomes by the Ragator (RAG) complex (141). mTOR is inhibited in amino acid deficiency. Supplementation of amino acids to regulate the mTOR signaling pathway and protein metabolism has received considerable attention as a potential treatment strategy for muscle atrophy after SCI (142–145). Amino acids activate the RAG complex by regulating the guanine dissociation and binding states of RAG. Activation of the RAG complex promotes the translocation and activation of mTORC1 to the lysosome (141, 146), thereby promoting the activation of mTORC1 and its downstream targets p70S6K and PHAS-1/4E-BP1, and thus enhancing synthesis of skeletal muscle protein (141).

The downstream target p70S6K of the PI3K/Akt/mTOR pathway can positively promote protein transcription: p70S6K is phosphorylated after activation and inhibits the negative regulator PHAS-1/4E-BP1 of the eukaryotic transcription initiation factor eIF4E (147). Release of this factor from the inhibitory complex allows it to bind to eIF4G and initiate translation, simultaneously promoting protein synthesis by prolonging the translation process (147) (Figure 4). Research has shown that 95% of the compensatory hypertrophic changes in muscle were blocked after rapamycin's specific inhibition of mTOR (137). In contrast, in unloaded muscles, the muscles showed a marked atrophic state, with significantly decreased phosphorylation levels of Akt protein and its downstream targets, and recovery of these when the load was restored. This suggests that phosphorylation of AKT and its downstream targets and activation of mTOR are required during muscle hypertrophy. Signaling pathways play a crucial role in mass recovery and hypertrophy of skeletal muscle atrophy caused by SCI (137, 148). The current study found that androgens and β2-adrenergic agonists have better therapeutic effects in activating IGF-1/PI3K/AKT/mTOC signaling pathway for skeletal muscle atrophy after spinal cord injury (19).

### Ubiquitin-proteasome system

The main component of skeletal muscle is myogenic fibers, and a characteristic of muscle atrophy is the rapid degradation of myogenic fibers (149). UPS in skeletal muscle is activated after SCI, the breakdown of proteins in skeletal muscle after SCI is mainly accomplished through the ubiquitin-proteasome pathway (50, 150), resulting in greater catabolism than anabolism in skeletal muscle (151), in which the degraded proteins are covalently linked to ubiquitin molecules *via* the ubiquitin ligase complex, which is then processed by the 26S protease. It has been shown that immobilization can up-regulate the mRNA expression of rat 26S proteasome (25, 50, 152, 153). As a critical pro-inflammatory factor, NF-κB, plays an important role between the inflammatory response to skeletal muscle atrophy after SCI and the balance of apoptotic and anti-apoptotic signaling, on the one hand, and determines whether cells will undergo apoptosis in response to other apoptosis-inducing-related factors such as TNF-α and TWEAK, affecting the proliferation and survival of skeletal muscle cells (83). However, the targets of NF-κB are ubiquitin-proteasome members, which can trigger ubiquitin molecular markers. The target protein is ultimately degraded by the ubiquitin-activating enzyme (E1), ubiquitin-conjugating enzyme (E2), and ubiquitin ligase (E3) (107, 154). Two E3 ligases, Atrogin-1 and MuRF1 play a vital role in the protein degradation process that mediates skeletal muscle atrophy (7, 107) (Figure 4), and their expression levels are upregulated in skeletal muscle after SCI (155). While the UPS is also key to the neural recovery of SCI (153). Gonzalez-Ruiz et al. found that the level of protein ubiquitination was significantly reduced after the application of epicatechin [(-)-epicatechi] in clinical SCI patients, the CSA of skeletal muscle was significantly increased; indicating that using UPS as a target of intervention can prevent skeletal muscle atrophy after SCI (156).

### Calpain

Calpain is the key enzyme in regulating apoptosis by mediating the degradation of cytoskeletal and membrane proteins, calpain is activated thus causing apoptosis of neural cells in the spinal cord and skeletal muscle cells in skeletal muscle after SCI (149, 157, 158), usually acting in conjunction with caspase-3 in the apoptosis of skeletal muscle, this is usually a key step in skeletal muscle atrophy (68, 151, 158, 159). There are studies that attenuate apoptosis after spinal cord injury from inhibition of calpain activation, and the current study found that SJA6017 (159), calpeptin, MDL-28170 could inhibit apoptosis by inhibiting calpain activity and have neuroprotective effects (158), but the effect on inhibiting skeletal muscle atrophy still needs to be further investigated.

### Autophagic lysosome system

Moderately activated autophagy helps protect cell structure and maintain normal cellular energy metabolism, and autophagic lysosomes are overactivated in skeletal muscle after SCI (160, 161). Beclin-1, a protein specific for autophagy, was found to be significantly elevated after SCI, resulting in the degradation of muscle proteins in skeletal muscle (160). Autophagy may depend on the regulation of mTOR signaling pathway (162), so it was found that regulation of mTOR may affect the autophagic process after injury, such as the application of AMPK inhibitors (163) or activators (164); also the autophagic process may not depend on mTOR, and inosito also have the effect of inhibiting autophagy (163). Exercise therapy is currently a very effective mainstream treatment for skeletal muscle atrophy, and studies have found that exercise training can slow down skeletal muscle atrophy by reducing autophagosome levels in skeletal muscle and thereby regulating autophagy (162).

### Myostatin

Myostatin is an essential member of the TGFβ family and acts as a negative regulator of skeletal muscle mass (20, 165). Some studies have found that its expression level may gradually increase with the inhibitory level of the PI3K-AKT signal after SCI, resulting in the loss of skeletal muscle mass (136, 166). It is a crucial muscle growth regulator, its inhibition increasing the mass of denervated skeletal muscle. In previous studies, myostatin-ActRIIB-ALK5-Smad2/3 was found to be an essential pathway affecting muscle mass (167) (Figure 4), and may be targeted at the molecular level. Previous studies have used synthetic myostatin inhibitors to interfere with Smad2/3, inhibiting the myostatin pathway (165) and delaying the skeletal muscle atrophy and metabolism caused by SCI (107, 168, 169) to treat post-SCI patients. The inhibition of myostatin has not been found to alleviate skeletal muscle atrophy of denervated limbs in experimental animals but has a specific therapeutic effect on disuse muscle atrophy, indicating that myostatin may be effective only in the presence of innervation (20). Studies have found (170) that the level of myostatin in the serum of patients with aerobic exercise, commonly used in the clinical treatment of SCI, increases to some extent over time. However, the mechanism underlying this increase needs to be further explored.

### Bone morphogenetic protein

BMP is a member of the TGFβ family and acts on a signaling pathway that affects the phosphorylation of Smad1, Smad5, and Smad8 proteins and ultimately converges on Smad4, thereby affecting muscle mass (Figure 4). BMP can inhibit muscle atrophy by binding to BMP-type receptor (ALK3). It can also negatively regulate the Fbxo30 (Musa1) gene, which plays a vital role in the regulation of the muscle atrophy-related ubiquitin-protease system (169, 171–173). Normal BMP signaling protects the neuromuscular junction (NMJ) and prevents excessive denervation of muscle fibers, and disturbed BMP signaling accelerates muscle atrophy (171, 174); BMP is expressed at low levels in motor neurons in a functionally intact state, and the expression of BMP2/4/7 and the corresponding ligands and receptors, phosphorylated Smad, are significantly upregulated in the spinal cord after SCI (175), thereby participating in the inflammatory response and neuronal apoptosis in the spinal cord, leading to abnormal neuromuscular signaling between the spinal cord and skeletal muscle, thereby affecting subsequent neurological recovery and skeletal muscle atrophy (173).

## Discussion

Skeletal muscle atrophy after SCI is a more complex and difficult clinical problem that requires multidisciplinary intervention. In recent years, little literature has outlined and analyzed the mechanisms of skeletal muscle atrophy after SCI. Therefore, this paper summarizes the pathophysiological changes of skeletal muscle atrophy after SCI. It analyzes the related factors and atrophy mechanisms, which can provide a specific theoretical basis and research direction for future clinical treatment to delay of skeletal muscle atrophy and enhance recovery of the patient's motor function. The current mainstream prevention and treatment methods are mostly exercise and hormone therapy. As described above in the article, exercise training and hormone therapy have shown relatively good therapeutic effects through various molecular pathways. The current clinical treatment for skeletal muscle atrophy after SCI is based on exercise training. For example, activity-based physical rehabilitation therapies (ABTs) (176–178) have shown sound therapeutic effects in improving neuromuscular plasticity and are widely used in many SCI patients for motor function training. They can enhance residual muscle strength (179), delay skeletal muscle atrophy, maintain and improve residual motor function, and improve patients' quality of life (180). More common forms of treatment include body weight-supported treadmill training, robot-assisted mobility training (8), heavy load strength training (HLT) (181), resistance training (RT) (182), but the therapeutic effect of ABTs decreases with the severity of the patient's injury. ABTs alone may therefore not be enough to promote muscle regeneration and motor function reconstruction in patients, and a combination of other adjuvant therapies may be needed (8, 176). In a review by Alvaro Megía García (183) et al. found that non-invasive transcutaneous spinal cord stimulation (tSCS) was effective in activating lower limb muscles, increasing muscle strength and improving muscle function; several studies (184, 185) found that the combination of N-3 unsaturated fatty acids and appropriate training delayed muscle atrophy and improved physical function. In addition, testosterone and androgen therapy can also significantly delay skeletal muscle atrophy (82).

However, these treatments often have their own limitations, such as hormone therapy has certain side effects, and exercise training is often difficult to achieve the desired results due to the lack of patient's endurance. SCI patients have energy and substance metabolism disorders (186), so a good nutritional intervention program may be able to improve the treatment effect of SCI skeletal muscle atrophy and optimize the treatment plan. Glutathione (GSH) is a small-molecule antioxidant substance that can counteract oxidative stress damage after SCI, thereby reducing skeletal muscle damage and protecting skeletal muscle from atrophy (68). Glycine is a non-essential amino acid as well as one of the main components of GSH. Glycine levels in serum, spinal cord and skeletal muscle tissue are decreased in SCI (187, 188), however glycine has been found to increase skeletal muscle mass, protect skeletal muscle functions under pathological conditions (189, 190), and fight against inflammatory response after disease (191). Leucine has also been found to be a potent amino acid effective in reducing skeletal muscle catabolism. The body's perception of leucine is impaired after injury due to inflammation and other factors, but glycine can restore the role of leucine in the body (192) and activates GSH metabolism (189). Glycine can be supplemented orally (193), therefore glycine therapy may be a safe, effective and promising dietary treatment.

Vitamin D has also been shown to be strongly associated with skeletal muscle health. Vitamin D can activate the vitamin D receptor in skeletal muscle cells by affecting the balance of calcium and phosphate, which promotes the proliferation and differentiation of skeletal muscle cells (194), and also affects the strength of skeletal muscle to a certain extent (195). The vitamin D content in the skeletal muscle of SCI patients is decreased (196), therefore, how to improve the skeletal muscle function of SCI patients through vitamin D supplementation has received a lot of attention from researchers in recent years (196, 197).

The metabolism of nutrients in the skeletal muscle of SCI patients is disturbed after the injury, and timely supplementation is needed. Therefore, appropriate dietary treatment and nutritional therapy are necessary to perhaps compensate for some of the drawbacks of medication and exercise training, and to improve the physical function of SCI patients by means of dietary intake, so that the therapeutic effects of multiple treatments can be maximized. However, how to carry out multiple nutrient supplementation, i.e., the ratio and content of each component nutrient, still needs to be clarified through further investigation of safe and effective nutritional intervention programs and the development of strict guidelines.

### Limitation

In order to write this review, we conducted a literature search in PUBMED, ISI Web of Science, and MEDLINE and Google Scholar databases before April 2022, searching “spinal cord injury,” “skeletal muscle,” and “atrophy” and so on as keywords. Although we conducted as extensive a literature search as possible and cited relevant and high-quality literature in our field whenever possible, there are still some relevant literature that may have been overlooked, as well as some ongoing studies and recent results that may not have been included.

## Conclusions

Multiple factors cause skeletal muscle atrophy after SCI, and the mechanism of atrophy is complex. The current clinical treatment methods involving drugs or exercise training are often insufficiently effective, especially for patients with more severe injuries. This review reveals that future therapeutic modalities may be explored and investigated at the cellular and molecular levels to optimize current clinical treatment options and improve the therapeutic effect of skeletal muscle atrophy after SCI, thus effectively promoting functional recovery after SCI. Besides, many regulatory factors related to skeletal muscle atrophy have a certain value in theory, but the relationship and influence between various regulatory factors also need to be further explored. Meanwhile, the development of regulatory means with clinical translational significance and how to carry out appropriate regulation are still an academic problem that needs to be further studied.

## Author contributions

Design and concepts: J-JL, D-GY, and XX. Definition of intellectual content: XX and ZT. Literature search: C-JZ, HK, HG, H-YD, and Y-ZP. Manuscript preparation: YY and Y-LJ. Manuscript editing: Y-LJ, L-JD, D-GY, and FG. Manuscript review: J-JL, D-GY, and FG. All authors approved the final version of the manuscript.
